# Breaking into nature's secret medicine cabinet: lichens – a biochemical goldmine ready for discovery

**DOI:** 10.1111/nph.70003

**Published:** 2025-02-26

**Authors:** Garima Singh, Francesco Dal Grande, Francis M. Martin, Marnix H. Medema

**Affiliations:** ^1^ Department of Biology University of Padova Via U. Bassi 58/B 35121 Padova Italy; ^2^ Botanical Garden University of Padova Via Orto Botanico 15 35123 Padova Italy; ^3^ Université de Lorraine, INRAE, UMR Interactions Arbre/Micro‐organismes, Centre INRAE Grand‐Est Nancy 54280 Champenoux France; ^4^ The National Key Laboratory of Ecological Security and Sustainable Development in the Arid Region, Northwest Institute of Eco‐Environment and Resources Chinese Academy of Sciences 730000 Lanzhou China; ^5^ Bioinformatics Group Wageningen University Droevendaalsesteeg 1 6708PB Wageningen the Netherlands

**Keywords:** bioactive metabolites, biosynthetic genes, drug discovery, lichenized fungi, natural products, omics, secondary metabolism, symbiotic fungi

## Abstract

Secondary metabolites are a crucial source of bioactive compounds playing a key role in the development of new pharmaceuticals. Recently, biosynthetic research has benefited significantly from progress on various fronts, including reduced sequencing costs, improved genome/metabolome mining strategies, and expanding tools/databases to compare and characterize chemical diversity. Steady advances in these fields are crucial for research on non‐modal organisms such as lichen‐forming fungi (LFF). Although most fungi produce bioactive metabolites, biosynthetic research on LFF (*c*. 21% of known fungi) lags behind, primarily due to experimental challenges. However, in recent years, several such challenges have been tackled, and, in parallel, a critical foundation of genomic data and pipelines has been established to accomplish the valorization of this potential. Integrating these concurrent advances to accelerate biochemical research in LFF provides a promising opportunity for new discoveries. This review summarizes the following: recent advances in fungal and LFF omics, and chemoinformatics research; studies on LFF biosynthesis, including chemical diversity and evolutionary/phylogenetic aspects; and experimental milestones in LFF biosynthetic gene functions. At the end, we outline a vision and strategy to combine the progress in these research areas to harness the biochemical potential of LFF for pharmaceutical development.

## Resumen

Los metabolitos secundarios constituyen una fuente esencial de compuestos bioactivos y, por ende, desempeñan un papel clave en el desarrollo de nuevos productos farmacéuticos. En los últimos años, la investigación biosintética se ha beneficiado significativamente de avances en diversos frentes, incluidos la reducción de los costos de secuenciación, el perfeccionamiento de las estrategias de minería genómica y metabolómica, y la expansión de herramientas y bases de datos para comparar y caracterizar la diversidad química. Estos progresos continuos son especialmente relevantes para el estudio de organismos no modelo, como los hongos formadores de líquenes (LFF, por sus siglas en inglés). Aunque la mayoría de los hongos produce metabolitos bioactivos, la investigación biosintética en LFF (que representan aproximadamente el 21% de los hongos conocidos) se encuentra rezagada, principalmente debido a desafíos experimentales. Sin embargo, en años recientes se han abordado varios de estos obstáculos y, de forma simultánea, se han generado bases de datos genómicos y herramientas críticas para valorizar este potencial. La integración de estos avances en la investigación bioquímica de los LFF ofrece una oportunidad notable para la obtención de nuevos descubrimientos. Esta revisión abarca los siguientes puntos: los avances recientes en las investigaciones ómicas de hongos y LFF, así como en quimioinformática; estudios sobre la biosíntesis de los LFF, incluyendo su diversidad química y aspectos evolutivos/filogenéticos; y hitos experimentales en la función de genes biosintéticos de LFF. Finalmente, se plantea una visión y estrategia para combinar estos desarrollos y aprovechar el potencial bioquímico de los LFF en la innovación farmacéutica.

## From nature to novel cures: the vital role of natural products

Natural products (NPs) are synthesized by plants, fungi, and bacteria as a part of both primary and secondary metabolism (Alur, 1999; Pott *et al*., 2019; Kuhnert & Collemare, 2022; Wang *et al*., 2022). Primary metabolites are essential for growth and reproduction, while secondary metabolites, also known as specialized metabolites, are critical for survival, defense, and ecological interactions (Demain & Fang, 2000; Bednarek & Osbourn, 2009; Kessler & Kalske, 2018; Pott *et al*., 2019; Erb & Kliebenstein, 2020; Gill *et al*., 2023). In recent years, the term specialized metabolites has gained preference over ‘secondary metabolites’, owing to their adaptive ecological functions and evolutionary importance in biotic and abiotic interactions. In this review, we will use the term secondary metabolite, given its widespread recognition and traditional usage in the scientific literature. Secondary metabolites (SMs) are well‐known for their diverse bioactive properties, which enable them to interact with biological systems and cells, and mediate biotic interactions of organisms. These bioactive properties are often pharmacologically beneficial to humans. The significant role of natural, bioactive metabolites in drug discovery is well‐documented, including their antimicrobial, antitumor, and anti‐inflammatory properties (Helaly *et al*., 2018; Hyde *et al*., 2019; Newman & Cragg, 2020; Miethke *et al*., 2021; Sun & Wesolowski, 2021; Grama *et al*., 2022).

The variety of SMs produced by an organism, encompassing different temporal and spatial scales and across the life stages of an organism, is referred to as chemodiversity (Müller *et al*., 2020; Müller & Junker, 2022). This diversity is evolutionarily shaped by an organism's interactions with abiotic and biotic factors, resulting in an enormous diversity of metabolites and corresponding biosynthetic genes in the genome, even among closely related species and genera (Müller *et al*., 2020; Sarrou *et al*., 2022). Overall, organisms possess a broader chemical potential than is evident in their metabolome at a particular time (Machado *et al*., 2017; Gavriilidou *et al*., 2022). Over the past few decades, a substantial portion (50–70%) of drugs have originated from either NPs or their analogs (Gould, 2016; Newman & Cragg, 2020; Grama *et al*., 2022). Interestingly, this vital contribution to the medical industry relies on only a few organisms, such as species and strains from the genera *Penicillium* and *Aspergillus*, whereas the vast majority of fungal taxa remains largely unexplored (Demain & Sanchez, 2009; Aly *et al*., 2011).

In recent decades, the demand for novel drugs has increased. Some of the reasons behind the pressing need are the emergence of new pathogens, rapidly evolving diseases, growing antibiotic resistance to prominent medicines and vaccines, the existence of incurable diseases, and the side effects or toxicity associated with existing drugs (Demain & Sanchez, 2009; Cragg & Newman, 2013). One way to meet this demand is to screen novel taxa for their bioactive potential and compounds for their bioactive properties.

In this review, we first discuss the factors that lead to the establishment of certain taxa as models for drug discovery, and then elaborate on why lichens, which are known as treasure chests of NPs, have not yet been utilized as drug leads. We discuss the chemical potential of these organisms as well as the state of the art of biochemical research on these organisms and elaborate on the key factors that hampered this field of research. We then provide a synthesis of how the technological advancements in recent years in genome sequencing, biosynthetic gene detection, and clustering pipelines can be amalgamated with advances in the field of lichen biochemistry and experimental research to exploit these taxa for drug discovery.

## Fungi and drug discovery: how we ended up playing favorites in fungal biosynthetic exploration

Fungi are prolific producers of bioactive SMs, and several approved drugs have traced their origins to fungi (Aly *et al*., 2011; De Silva *et al*., 2013; Demain, 2014; Hyde *et al*., 2019). Notable examples include cephalosporins (antibiotics, 18 700 billion USD; origin/inspiration: strains of the genus *Acremonium*), lovastatin (cholesterol‐lowering drug, 14 300 billion USD; isolated from *Aspergillus terreus* and *Monascus ruber*), and cyclosporins (immunomodulatory effects, 1990 billion USD; origin/inspiration: *Tolypocladium inflatum*) (Devi & Jayaseelan, 2020; Niego *et al*., 2023). These values refer to the gross income (Niego *et al*., 2023). Despite the vast diversity of fungi in nature, with *c*. 2.5 million species, and the second largest kingdom after animals, only a limited number have been tapped for their biosynthetic potential (Money, 2016; Niego *et al*., 2023). For instance, the genus *Penicillium* has been extensively exploited for bioactive metabolites and delivering/inspiring crucial medicines such as penicillin, griseofulvin, mycophenolic acid, mizoribine, and mevastatin (Pitt, 2014; Gaynes, 2017). Other intensively studied medicinal fungi include *Acremonium* spp., *Fusarium* spp., *Aspergillus* spp., and *Claviceps purpurea*. A recent review underscores this preference for drug leads by mapping fungal‐derived medicines onto the Ascomycota phylogeny, revealing that drug discovery efforts are predominantly concentrated on a few classes, for example Saccharomycetes, Eurotiomycetes (*Penicillium*, *Aspergillus*) and Sordariomycetes (*Acremonium* spp., *Claviceps* spp., *Melanocarpus* spp., *Ophiocordyceps* spp., *Tolypocladium* spp.) (Niego *et al*., 2023).

### Why have we extensively studied the secondary metabolites of few fungi?

Several practical factors have contributed to this pattern. Among these, ease of growing the organism in culture and maintaining pure cultures are the prime criteria for selecting taxa for drug leads. This was exemplified by the discovery of penicillin in 1929. Fleming studied *Staphylococcus* bacteria when *Penicillium* spores accidently germinated on a bacterial culture plate and killed all bacteria, revealing the antibiotic properties of penicillin (Fleming, 1929). To achieve a higher yield, other easily germinating *Penicillium* strains were hunted, eventually leading to the isolation of *P. chrysogenum* from a moldy cantaloupe, which produced six times more penicillin than the original Fleming strain (Chain *et al*., 2005; Gaynes, 2017). Effortless germination inspired controlled production, paving the way for drug development on an industrial scale in 1943.

**Fig. 1:**
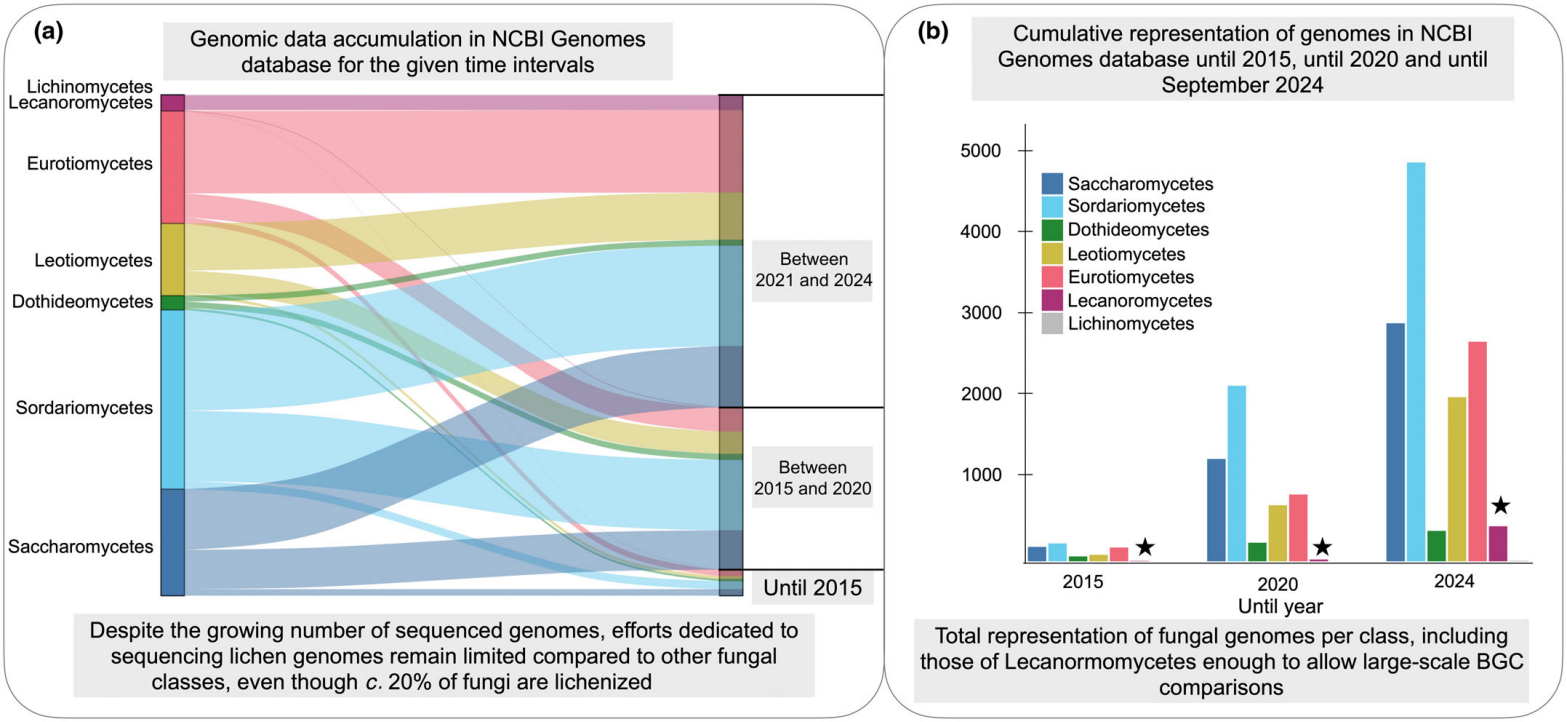
Global fungal genome sequencing efforts at specific time intervals (a) and cumulatively (b). Genome sequencing efforts have been ongoing for *c*. 20 yr, with a significant focus on fungal classes, particularly model fungi, such as Saccharomyces (yeast), Sordariomycetes (*Colletotrichum* spp., *Fusarium* spp.), and Eurotiomycetes (*Penicillium* spp. and *Aspergillus* spp.). Black stars indicate the bar for Lecanoromycetes in the plot, a group that primarily includes lichen‐forming fungi (LFF). Although LFF comprise *c*. 20% of all fungi and produce a wide array of bioactive metabolites, genome sequencing efforts for LFF have only gained momentum after the 2020s, resulting in the accumulation of *c*. 400 publicly available genomes to date. Even so, sequencing efforts per year for Lecanoromycetes remain low compared to other fungal classes. BGC, biosynthetic gene cluster. The number of Lecanoromycete genomes is indicated by stars.

The high industrial relevance of certain medicinally important genera is reflected in the genome sequencing efforts (Fig. 1a,b), which are driven by the need to improve strains for better cost‐to‐yield efficiency. Of the *c*. 4800 Ascomycota genomes in NCBI, *c*. 25% (±1200) belong to Saccharomycetes (e.g. *Saccharomyces cerevisiae*), followed by Eurotiomycetes with *c*. 600 genomes, including nearly 400 from *Penicillium* and *Aspergillus* (source: NCBI genomes/assembly database). By contrast, LFF – fungi engaging in symbiotic relationships with photosynthetic partners – algae, cyanobacteria, or both (DePriest, 2004; Lücking & Nelsen, 2018), which make up *c*. 20% of the fungal kingdom, represent only 2% of the sequenced Ascomycetes. Currently, roughly 100 LFF reference genomes are available, most of which have recently been sequenced (Fig. 1a,b; source: NCBI genomes/assembly database).

## Uncovering nature's secrets: charting new territories for chemical exploration

### Lichenized fungi

The lichenized lifestyle is mostly found in Ascomycota, with over 99% of all lichenized fungi being found in Ascomycota (Cousin, 2014; Lücking & Nelsen, 2018). Within Ascomycota, the largest lichenized clade is Lecanoromycetes, which contains 78% of all lichens, followed by Arthoniomycetes (8%), Eurotiomycetes (6.5%), Dothideomycetes (4%), and Lichinomycetes (2%) (Cousin, 2014; Lücking & Nelsen, 2018). Of these, only Lecanoromycetes, Arthoniomycetes, and Lichinomycetes are almost exclusively lichenized. The fungal partner, known as the mycobiont or lichen‐forming fungus, defines the name of the lichen, whereas the photosynthetic partner is known as the photobiont.

### The state of lichen biochemical research

**Fig. 2:**
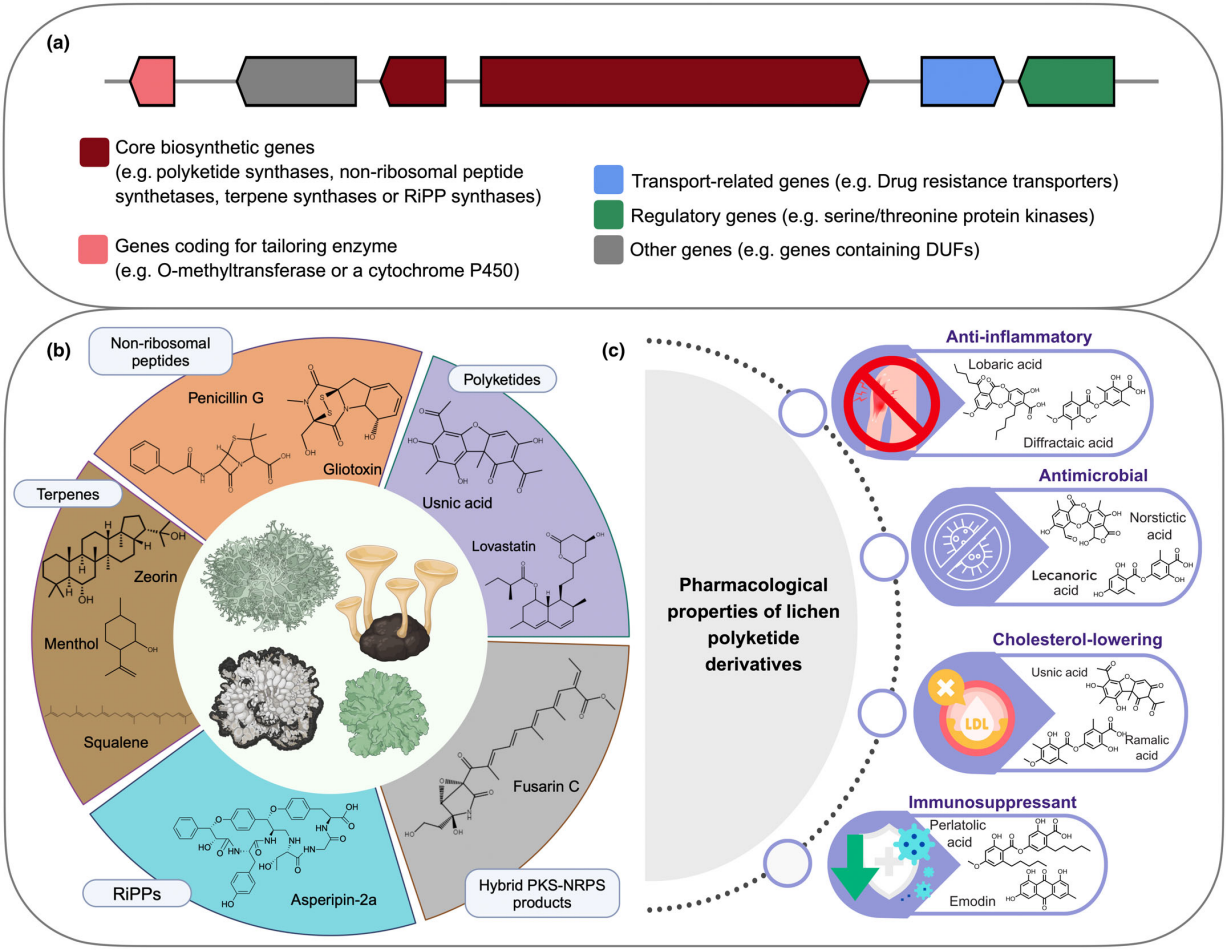
Typical organization of a fungal biosynthetic gene cluster (BGC), predominant metabolite types in lichen‐forming fungi (LFF), and the bioactive properties of some common polyketide derivatives. (a) An example of a BGC, as shown in the presented figure, as predicted by AntiSMASH. A cluster is composed of one or more core genes, along with genes coding for tailoring enzymes, transport‐related proteins, and regulatory elements. Core genes encode the backbone of the compound, while tailoring genes modify this basic structure to produce the final molecule. (b) Common BGCs in LFF and some of the compounds they encode. Polyketides, including their derivatives, are synthesized by polyketide synthases (PKSs) and are the most predominant BGC class in LFF (*c*. 50%) (Kim *et al*., 2021; Singh *et al*., 2022). PKS derivatives are among the most extensively studied compounds from non‐LFF and LFF with respect to their molecular structures, synthesis pathways, regulatory mechanisms, and bioactivity. (c) Bioactive properties of some well‐studied PKS derivatives. The bioactive properties of PKS derivatives have been widely demonstrated, highlighting their potential in pharmaceutical and biotechnological applications. This figure was partially created in BioRender (https://BioRender.com/p88a746).

Lichens are considered a treasure chest of biosynthetic genes and bioactive NPs (Fig. 2a–c). Most lichen metabolites are species‐specific or are synthesized by a few taxa. To date, *c*. 1000 lichen‐related substances have been characterized, many of which are species‐specific and have diverse pharmacological activities (Goga *et al*., 2018). These substances exhibit a range of properties, including antibiotic, antimycobacterial, antiviral, anti‐inflammatory, analgesic, antipyretic, antiproliferative, and cytotoxic effects (Fig. 2c). A single lichen species can often secrete multiple metabolites, each with distinct bioactivity (Torres‐Benítez *et al*., 2017; Ingelfinger *et al*., 2020; Sepúlveda *et al*., 2022).

**Fig. 3:**
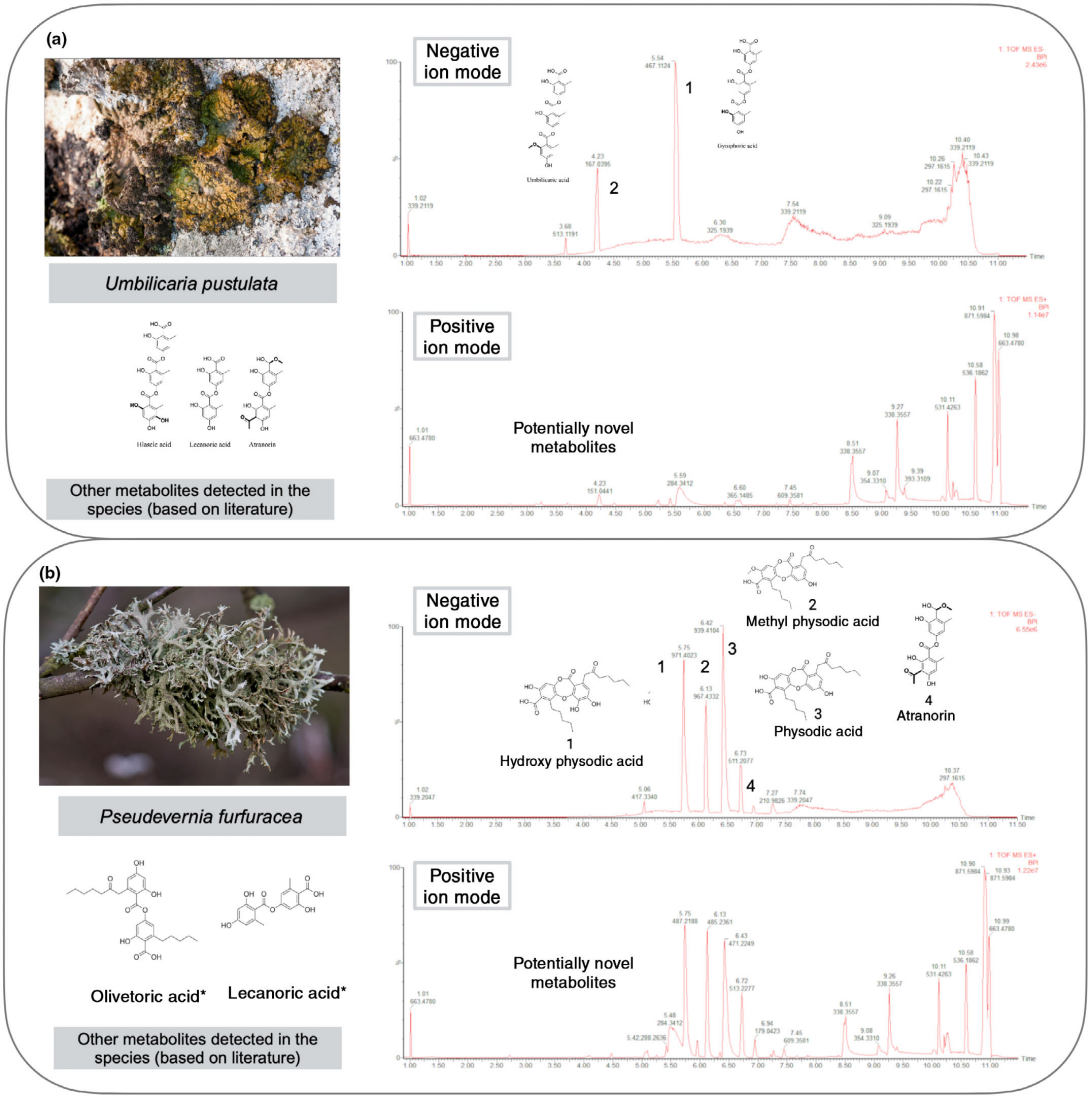
Mass spectra of two common lichen‐forming fungi demonstrating a plethora of unidentified compounds. (a, b) showcase the mass spectra of *Umbilicaria pustulata* and *Pseudevernia furfuracea*, respectively (please see Supporting Information Notes S1 for details). Both species produce a range of bioactive metabolites (Türk *et al*., 2006; Kello *et al*., 2021), along with numerous yet‐uncharacterized compounds, as indicated by the MS peaks in positive ion mode. This figure highlights the enormous unexplored metabolic potential of lichens. Metabolites marked with stars are reported from a different chemotype of this species but not in this MS run.

On average, the genome of a lichenized fungus contains *c*. 40–50 biosynthetic loci (genes, or sets of genes, organized collinearly on the genome and involved in the synthesis of one or more secondary metabolites; Fig. 2a), of which *c*. 35–50% belong to the polyketide synthases (PKS) class (Gerasimova *et al*., 2022; Singh, 2023). PKS derivatives have gained significant attention due to their stability and detectability through techniques such as TLC and HPLC, aided by their high molecular weight and abundance. Advanced methods such as LC‐MS and UV‐HPLC have revealed a greater diversity of these compounds (Olivier‐Jimenez *et al*., 2019; Sepúlveda *et al*., 2022; Singh *et al*., 2022; Fig. 3a,b). While nonribosmal peptide synthetases (NRPSs), terpenes, and ribosomally synthesized and post‐translationally modified peptides (RiPPs) also contribute to the biosynthetic landscape of LFF, few NRPS‐ and terpene‐related compounds are known, and no ribosomal peptides have been reported.

### What is the problem? Technical challenges in deorphanizing biosynthetic genes

Despite the rich metabolic diversity of LFF, studies linking LFF secondary metabolites to their genes are scarce (Singh, 2023). As an obligate symbiosis (for the fungal partner; the photosynthetic partners can also be facultative and are reported to be free‐living as well), it is difficult to grow the mycobiont in isolation as pure cultures and to perform gene knockout or knockin experiments (Stocker‐Wörgötter, 2001, 2008; Muggia *et al*., 2017). The complex lifestyle of lichens poses significant challenges in generating high‐quality genomes of fungal partners. As symbiotic associations, lichens consist of multiple partners, making it difficult to disentangle the genomes of the fungal and photobiont components. Furthermore, the slow growth of lichens, coupled with the difficulty in cultivating the fungal partner independently, limits the availability of pure and sufficient DNA for sequencing, thereby complicating genomic studies. In addition, the slow growth rate of LFF and the fact that the stimuli triggering secondary metabolite synthesis are either unknown or may not be reproducible in the laboratory further hinder NP‐related characterization in lichens.

#### The biggest bottleneck: culturing and heterologous expression of genes

Although the bioactivity of crude extracts has been well‐documented for numerous lichen species for several decades, the genetic and molecular pathways involved in lichen metabolite synthesis remain unclear. This is primarily because lichen‐forming fungi either do not grow in isolation (without their symbiotic photobiont) in culture or exhibit extremely slow growth, making the experiments time‐intensive. To date, only a few lichen‐forming fungi have been successfully cultivated under laboratory conditions, and consequently, biosynthetic studies have been predominantly limited to a few culturable model LFF such as *Usnea* spp. and *Cladonia* spp. Even in these extensively studied model LFF, establishing connections between specific molecules and their corresponding genes is a protracted process, and a definitive link via heterologous expression remains elusive (Stocker‐Wörgötter, 2001; Bertrand & Sorensen, 2019). In an attempt to heterologously express usnic acid genes, the candidates were transcribed, but not translated, precluding conclusive links between genes and metabolites (Bertrand & Sorensen, 2019). Subsequently, this link was established, and the usnic acid gene cluster was recently identified through genome sequencing and PKS gene analysis (Abdel‐Hameed *et al*., 2016; Pizarro *et al*., 2020; Egbert *et al*., 2022). Additionally, while the evolutionary relationships of genes involved in NP synthesis could potentially facilitate the association of molecules with genes, the fundamental background for persuing this, i.e. open‐access genomes, and robust phylogenies, particularly for key biosynthetic classes, such as PKSs and NRPSs, were not available until recently.

## Why now?

**Fig. 4:**
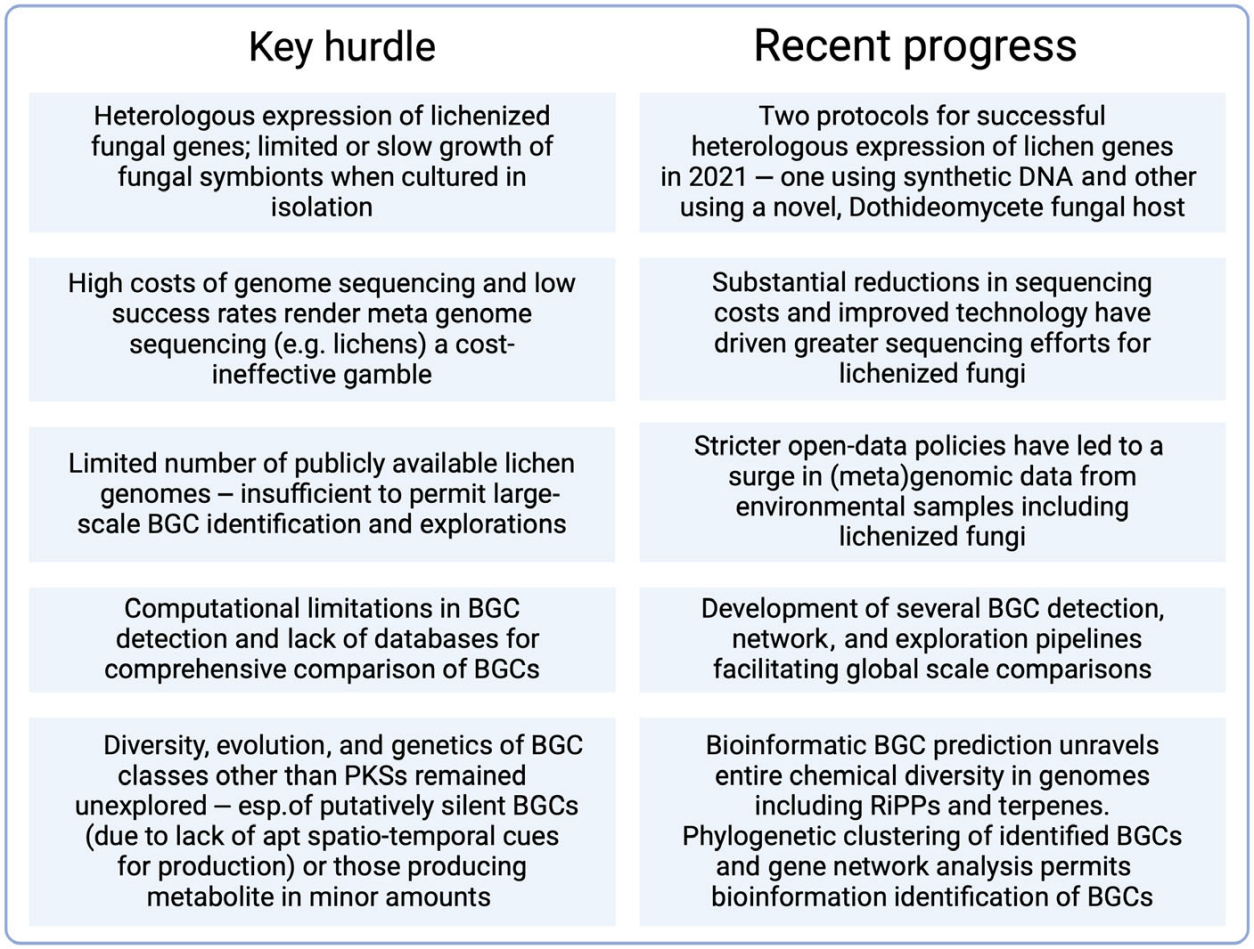
Box showing the key challenges in lichen experimental biochemistry and recent technological advances that help overcome or mitigate these challenges. This figure was created in BioRender (https://BioRender.com/l78b041).

Over the past few years, substantial advancements have been made across various domains, including biological and chemoinformatic techniques for identifying and grouping NP structures and biosynthetic genes, methods for genome assembly using open‐data practices, improvements in metabolite detection and expression, and experimental validation (Fig. 4). By integrating these developments from diverse fields, we can connect the dots on the horizon, unveiling new opportunities to expand the scope of lichen biochemical research.

### The first dot – crucial milestones for navigating the biosynthetic landscape

#### The potential of genome mining: a continuing genomic revolution – equation of expense and duration

Genome sequencing has witnessed remarkable progress, leading to substantial reductions in both the financial and time requirements. For example, the initial human genome sequencing project spanned 13 yr and required *c*. $3 billion. By contrast, contemporary Illumina sequencing techniques cost between 6€ and 10€ per gigabase and can be completed within days. Long‐read sequencing methods, although slightly more expensive at €33 to €35 per gigabase, can produce results in only a few hours. These technological advancements have been particularly advantageous for smaller genomes, such as LFF, which typically have an average nuclear genome length of 50 Mb. In addition, it is now possible to experimentally enrich and computationally isolate reads from lichen‐associated organisms, including algae and bacteria, thereby facilitating the study of their interactions. Furthermore, several global initiatives aim to build a genomic database of organisms. For instance, the Earth BioGenome Project (EBP), including the European Reference Genome Atlas (ERGA), aims to produce a database of high‐quality reference genomes of *c*. 1.5 million eukaryotic species (McCartney *et al*., 2024). Although the genomes under these initiatives were generated for different aims, they constitute a valuable resource for spin‐off studies and could be mined for many important discoveries.

#### DNA sample specifications

Sequencing technologies have become less demanding in terms of sample preparation as well as the quantity and quality of input DNA. Although cell lines were once the preferred biological material for sequencing, the scope has now expanded to include complex environmental samples, including associated microorganisms such as bacteria and viruses. This shift has been made possible by the development of metagenomic binning pipelines, which allow for the taxonomic separation of reads and contigs after sequencing. This advancement has had profound implications for genomic research on complex organisms involved in symbiotic associations, for which obtaining pure cultures has been laborious, time‐consuming, and often successful in only a limited number of species.

#### Data‐sharing regulations

A significant milestone is the development and partial implementation of an open‐data policy that promotes publishing and makes genomes accessible once a study is under review or ready for publication. Many journals support open‐data policies and adhere to FAIR (Findable, Accessible, Interoperable, and Reusable) principles, requiring or urging authors to deposit their data in well‐known repositories, such as dedicated databases at the NCBI, EBI, and JGI (e.g. GenBank), or general long‐term data storage solutions such as Figshare, Zenodo, and DataDryad. The policy has garnered strong support, enhanced data‐intensive science and prevented redundancy and resource waste.

#### The collateral benefits of genome mining

This approach allows for comprehensive characterization of an organism's biosynthetic potential, including active, silent, and temporally or quantitatively limited biosynthetic gene clusters (BGCs) (Osbourn, 2010; Chen *et al*., 2017). Advances in high‐throughput sequencing (HTS), particularly long‐read sequencing, have improved the resolution by providing a higher N50 (in the order of Megabases), enabling the sequencing of long genes and extensive gene clusters. Currently, *c*. 100 LFF genomes are available in NCBI. Although limited compared with other fungal classes (Fig. 1a,b), these genomes cover diverse taxa and habitats, offering insights into the biosynthetic potential of LFF and aiding in the discovery of NPs.

#### Updated BGC detection and comparison pipelines

Several databases have been established to improve the identification and comparison of biosynthetic diversity across different life domains. One such example is the MIBiG database, which contains 7603 validated and annotated BGCs. This resource provides extensive information on cluster composition as well as the structures, bioactivity, and biosynthesis of associated metabolites (https://mibig.secondarymetabolites.org; Zdouc *et al*., 2024). The MIBiG database is linked to antiSMASH (Blin *et al*. 2023), enabling efficient BGC detection and providing insights into potential metabolite diversity. Clustering algorithms such as BiG‐SCAPE (Navarro‐Muñoz *et al*., 2020) and BiG‐SLiCE (Kautsar *et al*., 2021b) can group antiSMASH‐predicted BGCs and reference BGCs from MIBiG into families based on similarity, to rapidly annotate which BGCs are likely associated with biosynthetic pathways for the production of known compounds (or structurally closely related ones). The BiG‐FAM (Kautsar *et al*., 2021a) database, which comprises 1225 071 BGCs from sources such as GenBank, RefSeq, and metagenomes, organizes these BGCs into 29 955 gene cluster families precomputed using BiG‐SLiCE (https://bigfam.bioinformatics.nl/home). This organization supports the exploration and comparison of homologous BGCs and is compatible with antiSMASH, providing immediate insights into the biosynthetic landscape of newly sequenced taxa compared to publicly available data.

In addition to these general resources, specialized databases also address specific needs. For example, ARTS targets the identification and prioritization of BGCs encoding novel antibiotics by detecting antibiotic‐resistant targets, offering precomputed results for over 70 000 bacterial genomes and MAGs (https://funarts.ziemertlab.com; Mungan *et al*., 2022; Yılmaz *et al*., 2023). Together, these resources not only enhance our ability to analyze and predict the biosynthetic potential of various organisms but also support the study of unconventional and nonmodel taxa, such as lichens, by providing a comprehensive suite of tools for large‐scale data exploration and comparison.

### The second dot – stabilizing evolutionary relationships of the biosynthetic genes

#### Non‐reducing polyketide synthases evolution and stabilization of phylogenetic relationships

Fungal PKS phylogenies are constructed using sequences from the KS or PT domains and sometimes the entire non‐reducing polyketide synthases (NR‐PKS) gene. Initial PKS phylogenies identified three main groups: one exclusively bacterial and two exclusively fungal groups. The fungal clades included a reducing PKS clade and a non‐reducing PKS clade, with the latter divided into subclades I (toxins and non‐melanin pigments), II (melanin), and III (uncharacterized, with a CMeT domain after the ACP domain). As new genomes were sequenced, NR‐PKS phylogeny expanded. Subclades I and II were further divided into five groups (I–V) based on cyclization regioselectivity and compound size, whereas subclade III was split into two groups (VI and VII) for aromatic compounds (each clade representing a different compound as orsellinic acid derivatives, melanins, melliolides, aflatoxin/fusarubin, and anthraquinones). Recent studies have introduced groups VIII and IX for basidiomycete fungi and β‐orcinol compounds from lichenized fungi, respectively (Pizarro *et al*., 2020; Kim *et al*., 2021; Singh *et al*., 2022). Group VIII contained NR‐PKSs from both basidiomycetes and ascomycetes, however, while ascomycete NR‐PKSs were found in all groups, and basidiomycete NR‐PKSs were restricted to group VIII. The most current PKS phylogeny includes 11 groups, each with several PKSs involved in the synthesis of structurally unique compounds. Although phylogenetic clustering alone cannot definitively link a gene to a molecule, it predicts the most likely PKS for a given compound.

#### PKS derivatives (clustering‐based prediction of potential compounds)

Despite the long‐known bioactive potential of lichen metabolites, we have only recently started to understand the genetics behind NP synthesis. The first PKS linked to a NP was isolated from *Cladonia grayii* in 2011 (Armaleo *et al*., 2011). Since then, a few metabolites have been linked to their gene clusters, mostly through an integrative approach using chemoinformatics, phylogeny, and molecule‐to‐gene correlations. The spin‐off benefit of these linking efforts was that the annotation of PKS phylogeny continued to expand. In addition to the main clade of interest, all PKSs from LFF can be placed on a phylogenetic tree, unraveling the putative metabolic potential of the organisms, pertaining to PKS‐derivatives. This potential may have gone undetected owing to quantitative, seasonal, or temporal variations in compound synthesis. For instance, many lichens contain PKS16, which is involved in the synthesis of orcinol compounds, although the corresponding compound remains unknown to date.

In the process of identifying PKS16, which codes for the biosynthesis of orcinol‐depside olivetoric acid in *Pseudevernia furfuracea*, atranorin PKS (PKS9) was also identified based on the phylogenetic clustering of a *P. furfuracea* PKS within the PKS9 clade (Singh *et al*., 2021).

#### Beyond PKSs

Polyketide synthases, NRPSs, terpene synthases, and RiPPs are the primary components of the LFF biosynthetic landscape. Among these, only PKSs have been successfully expressed in heterologous systems (Kealey *et al*., 2021; Kim *et al*., 2021). A recent genome mining study across all reference LFF genomes focused on terpenes, which are another biosynthetic class (Singh *et al*., 2024). The analysis revealed that two families of terpene BGCs, likely squalene/phytoene synthase BGCs, are conserved across LFF, whereas the others are unique to specific taxa. Of the 5–10 terpene BGCs typically found in LFF genomes, only one is widely shared, whereas the rest are rare and often taxon‐specific. Some terpene derivatives including diterpenoids and triterpenoids have been identified in LFF. For example, zeorin, produced by numerous LFF, exhibits antioxidative and anti‐inflammatory properties. Currently, no evolutionary or phylogenetic analyses are available. Although terpene BGCs account for *c*. 20% of the total biosynthetic capacity of LFF, their evolution, and diversity remain largely unknown.

### The third dot – advances in lichen biochemical research

#### LFF chemical space: no longer searching for a needle in a haystack

**Fig. 5:**
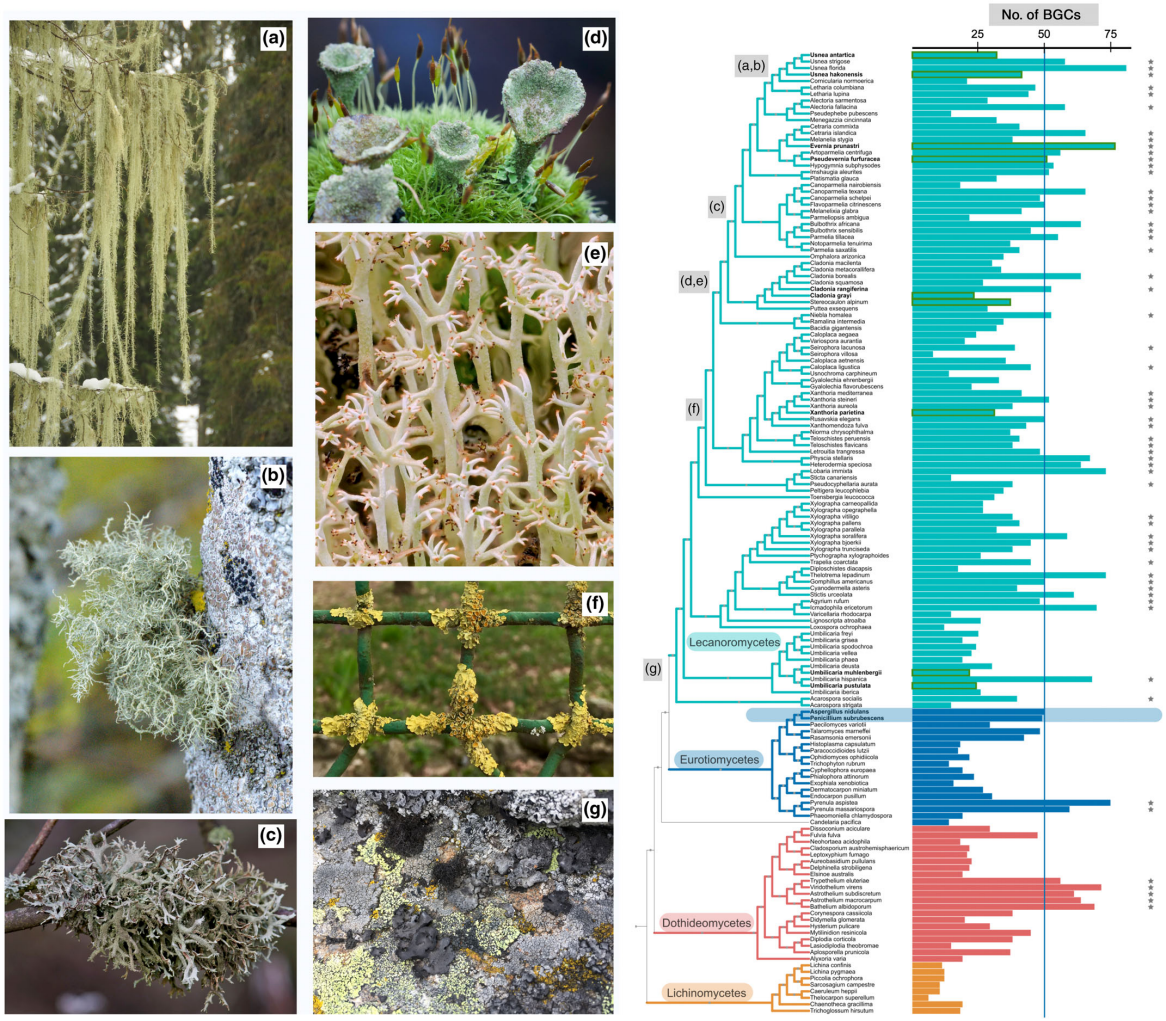
Phylogenetic position of some of the most biochemically studied lichens. (a) *Usnea longissima*, (b) *Usnea* sp., (c) *Pseudevernia furfuracea*, (d) *Cladonia* sp., (e) *Cladonia rangiferina*, (f) *Xanthoria parietina*, and (g) *Umbilicaria* spp. The phylogenomic tree was constructed using the genomic data from 102 lichen‐forming fungi (LFF) and 47 non‐lichenized fungi from the closely related classes Eurotiomycetes, Dothideomycetes, and Lichinomycetes (Supporting Information Table S1; Notes S1, S2). The colored horizontal bars indicate the total number of biosynthetic gene clusters (BGCs) per taxon. The blue vertical line indicates the highest number of BGC in *Penicillium* and *Aspergillus* spp. (indicated by the blue box). Lichen‐forming fungi with more BGCs than the modal taxa are marked by grey stars. This figure illustrates that many LFF exhibit greater biosynthetic potential than model fungi commonly used in drug discovery. Furthermore the most BGC‐rich LFF still remain underexplored in terms of their metabolic diversity. Part of this figure was created in BioRender (https://BioRender.com/n44w847).

Owing to large‐ and small‐scale genome mining efforts, our understanding of the evolution of LFF chemical diversity, biosynthetic genes, and metabolites has greatly expanded in recent years. More genomes are becoming publicly available every day, enabling comparative genomics of biosynthetic genes and providing us with a broad overview of LFF chemical space, including the approximate number of BGCs per taxon and the most common and rare BGC classes. We also know which genera and families have relatively fewer BGCs, such as *Umbilicaria* (Umbilicariaceae; *c*. 15–25 BGCs per species) and which is particularly rich, such as *Evernia prunastri* (Parmeliaceae, *c*. 75 BGCs) (Fig. 5).

A general overview of the biosynthetic space in an organism not only helps us to identify structurally and potentially functionally rare metabolites but also the evolution of the organism's chemical potential in relation to its ecology and lifestyle. For instance, given the potential role of RiPPs as mycotoxins (Chooi & Kessler 2022), the presence of a high number of RiPP BGCs suggests a competitive advantage against lichenicolous or other parasitic fungi. The availability of genomes and biosynthetic comparison pipelines/databases facilitates informed decisions when linking genes to molecules and narrowing down candidates for characterization. For instance, this approach aided in the characterization of depside PKS from *P. furfuracea* (Kealey *et al*., 2021). In this case, the candidate PKS was identified in *Pseudevernia furfuracea* through a comparative genomic approach (Calchera *et al*., 2019). Another team of researchers used synthetic DNA, expressed it in a heterologous host, and obtained lecanoric acid, a common PKS‐derived compound with broad‐spectrum bioactivities (Kealey *et al*., 2021).

#### The suitable host

In recent years, there have been significant advancements in the field of LFF research, including successful heterologous expression of PKSs. Notably, lecanoric acid PKS and atranorin PKS were expressed in two different hosts: yeast (Kealey *et al*., 2021) and the plant pathogenic fungus, *Ascochyta rabei* (Kim *et al*., 2021). However, yeast may lack the necessary starter unit specific to fungi, leading to different metabolites compared with native LFF. For example, when olivetoric/physodic acid PKS from *P. furfuracea* is expressed in *S. cerevisiae*, it produces lecanoric acid, likely due to the absence of an appropriate starter unit (Kealey *et al*., 2021). By contrast, the expression of atranorin PKS in *A. rabei* successfully yielded the desired compound. Another promising host is *Aspergillus oryzae*, in which two prenyltransferases from *Ramalina intermedia* and *Acarospora strigata* were expressed and identified as 4‐O‐dimethylallyltyrosine synthases (Iacovelli *et al*., 2024). These studies underscore the importance of selecting an appropriate host to validateLFF biosynthetic genes.

**Fig. 6:**
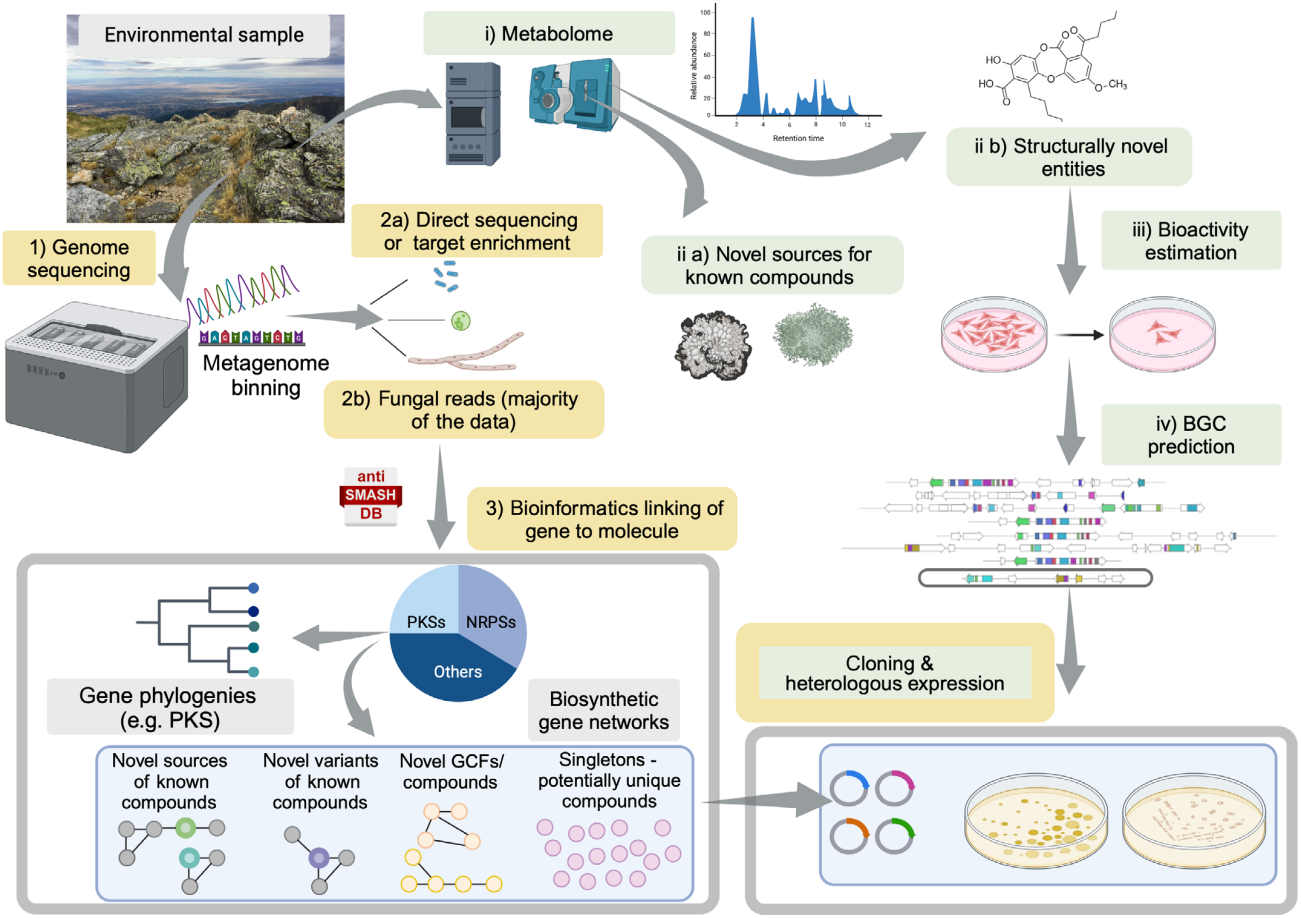
A framework for schematic study, comparison, and exploitation of lichen‐forming fungi (LFF) chemodiversity. Arrows indicate the direction of workflow steps, with bifurcated arrows representing potential or alternative possibilities. The pipelines, indicated by orange and green boxes, delineate the genotype‐up and phenotype‐down approaches, respectively. Fresh environmental samples can be collected and sent for genome sequencing (genotype‐up approach) and parallel metabolomic analysis (phenotype‐down approach), without the need for pure cultures. The resulting reads are obtained from the lichen metagenome, predominantly containing fungal reads but also from algae, bacteria, and viruses (genotype‐up approach). These reads are binned, either before or after assembly, to isolate the fungal genome. After several rounds of contaminant removal and purity assessments, the resulting fungal genomes are subjected to downstream analysis, including identification of biosynthetic genes, phylogeny construction, and similarity network generation to identify known, novel, and unique biosynthetic gene clusters (BGCs). These BGCs can then undergo targeted downstream analyses such as cloning and heterologous expression to validate the compounds they encode. Additionally, BGC data can be used to correlate genes with metabolites to estimate the most likely BGC for a natural product. The resulting candidate BGCs can then be characterized by heterologous expression to establish definitive connections. In the phenotype‐down approach the samples are subjected to metabolite detection following by bioactivity estimation, BGC prediction and heterologous expression of genes of interest. The last step, cloning and heterologous expression, is indicated by a green box with an orange border and is common to both approaches. PKS, polyketide synthases; NRPS, nonribosmal peptide synthetases; GCF: Gene Cluster family. This figure was created in BioRender (https://BioRender.com/m76z830).

Within a cluster, a combination of expressed genes leads to the production of structurally and functionally distinct molecules. Heterologous expression allows researchers to experiment with different gene combinations, not only to understand the role of each gene in a pathway but also to explore the plasticity of the cluster in producing various compounds (Fig. 6). For instance, a single PKS may produce a backbone depside, which may be the final product; for example, lecanoric acid, or olivetoric acid, or could be modified with the help of a tailoring enzyme to produce another compound; e.g., olivetoric acid is oxidized to physodic acid with the help of CYP450, which is present alongside PKS in the cluster (Singh *et al*. 2021). Similarly, while gyrophoric acid is the product of PKS, it may undergo downstream oxidation, methylation, or both to produce umbilicaric acid and hiascic acid (Singh *et al*. 2022). A cluster can be manipulated in the host to favor the production of a molecule with the desired structure and bioactivity by selecting specific combinations of genes.

#### Decoding PKS BGCs: unveiling BGC variations and corresponding modifications in metabolites

With the increasing availability of LFF genomes in public databases, researchers have begun to utilize this information to construct PKS phylogenies and to identify relevant PKSs in newly sequenced genomes. For instance, Pizarro *et al*. (2020) leveraged these data to pinpoint the usnic acid clade and examined variations within clusters across various LFF. In a similar manner, Llewellyn *et al*. (2023) used this information to investigate the evolutionary history of melanin PKSs. The process of metagenomic identification of all PKSs within an organism, followed by the construction of a phylogenetic tree based on characterized PKSs, aids in identifying potential genes for specific compounds. Established gene‐to‐molecule connections for lichen PKS include grayanic acid (PKS16), lecanoric acid (PKS16), olivetoric acid, physodic acid (PKS16), gyrophoric acid (new, sister clade to PKS16), atranorin (PKS23), melanins (PKS5), and usnic acid (PKS8). To confirm these connections, candidate genes can be validated in appropriate hosts, thereby establishing a definitive link between the genes and metabolites.

## Connecting the dots: the way forward

How can a growing wealth of genomic resources be leveraged? The extensive availability of genomes facilitates the comparison, clustering, and analysis of diverse BGCs. Lichenized fungi typically harbor 20–50 biosynthetic genes in their genomes (Fig. 5). However, only a few metabolites have been detected in a single taxon. Sensitive techniques, such as MS and UV‐HPLC, may even detect the metabolites produced in lower amounts,unraveling more BGCs as active than previously thought, i.e, to up to 10 instead of two to five (Torres‐Benítez *et al*., 2017; Singh *et al*., 2022; Singh, 2023). Yet many BGCs are still orphan or silent. Genome mining is an effective tool for uncovering widely distributed and evolutionarily conserved clusters, including the enzymes with putatively unknown conserved domains but vital functions, even if automated pipelines fail to recognize them. The combination of genome mining and network‐based approaches can uncover conserved pathways, which could then be tested for functionality and used to infer their ecological roles and evolutionary histories. Although this practice has not yet been widely applied to LFF, it has revealed important biosynthetic pathways in other organisms.

For example, a recent study detected a homologue of Pyr4‐family terpene cyclases in *c*. 2000 fungal genomes. This prompted the researchers to characterize this pathway, eventually uncovering fungal onoceroid triterpenoids, and novel onoceroid synthases and sesquiterpene hydroquinone (Tang & Matsuda, 2024). Similarly, in strains of the bacterium *Salinispora*, molecular networking and pattern‐based genome mining revealed known compounds and their derivatives, as well as new compounds. This approach led to compound‐gene cluster pairings and the linking of clusters to the characterization of quinomycin‐type depsipeptide retimycin A (Duncan *et al*., 2015). In case of actinobacteria, the combination of targeted mining and network‐based analysis revealed the widespread distribution of macrolactam BGCs, a structurally diverse group of bioactive natural products derived from polyketides, known for their exceptional pharmacological properties, including antibiotic, antifungal, anticancer, and immunosuppressant activities (Seibel *et al*., 2023). We expect the same scenario for LFF and advocate the large‐scale application of genome mining and network approaches for organizing the LFF BGC space into widespread or unique pathways and unraveling novel pathways. Biosynthetic and network‐based detection of widely distributed pathways in lichens, followed by characterization, could reveal the crucial ecological functions and evolutionary histories of their secondary metabolites, an aspect that remains largely speculative to date.

In Fig. 6, we delineate the workflow for the systematic screening of novel taxa for their bioactive potential. We propose a standardized, broad‐scale, and efficient method for exploring biosynthetic gene diversity by integrating technological advances to reduce labor, while simultaneously streamlining dereplication and avoiding redundant rediscovery. This approach encompasses the biosynthetic linking and clustering of identified BGCs to determine the most promising BGCsas potential drug leads. Bioinformatic identification and categorization of detected BGCs into known, unknown but common, or unknown and rare enables researchers to prioritize those with the highest potential for drug discovery (Fig. 6). In conclusion, by integrating diverse research frontiers, we can effectively harness the vast bioactive potential of BGCs from non‐model, biosynthetically rich organisms, avoid dereplication, and identify novel and unique candidates for targeted drug discovery.

## Competing interests

MHM is a member of the scientific advisory board of Hexagon Bio.

## Author contributions

GS, FDG, FMM and MHM designed the article and performed the research. GS did data analysis, collected and interpreted the data. GS, FDG, FMM and MHM wrote the manuscript.

## Disclaimer

The New Phytologist Foundation remains neutral with regard to jurisdictional claims in maps and in any institutional affiliations.

## Supporting information


**Notes S1** Methods and results of the MS analysis and phylogenetic tree reconstruction.

**Notes S2** Concatenated alignment used to infer the phylogenetic tree. This file can be opened in BBEdit for Mac users, or in Notepad++, Sublime Text, or Word for Windows users and can be visualized in alignment programs as Geneious or Mafft.

**Table S1** Voucher information of the samples.
Please note: Wiley is not responsible for the content or functionality of any Supporting Information supplied by the authors. Any queries (other than missing material) should be directed to the *New Phytologist* Central Office.
