# Supplementary material for: Breaking into nature's secret medicine cabinet: lichens – a biochemical goldmine ready for discovery
Source: New Phytol. 2025 Feb 26;246(2):437–49. doi: 10.1111/nph.70003 (PMC11923402; doi:10.1111/nph.70003)
Supplement: Supplementary file 1 — Notes S1 Methods and results of the MS analysis and phylogenetic tree reconstruction. [file NPH-246-437-s003.pdf]

New Phytologist Supporting Information

Article title: Breaking into Nature's Secret Medicine Cabinet: Lichens, a Biochemical Goldmine Ready for Discovery

Authors: Garima Singh, Francesco Dal Grande, Francis Martin, Marnix H. Medema

Article acceptance date: 18 January 2025

Notes S1 Methods and results of the MS analysis and phylogenetic tree reconstruction.

Methods:

Metabolite profiling and molecular networking

To identify potential depsides and depsidones in the lichen extracts, we conducted mass spectrometry (MS) analysis in negative ion mode on two samples. For each specimen, lichen thallus materials (ca. 15 mg) were grinded into powders under liquid nitrogen. Metabolites were extracted three times from grinded powers with acetone (800 µL each time), and then the extracts were combined and evaporated. Dried residues were re-constituted in 2 mL solvent mixture of methanol and acetonitrile (50:50, v/v), and a 50 µL aliquot was diluted 20 times with the same solvent mixture and filtered (0.2 µm, PTFE) before liquid chromatography-mass spectrometry (LC-MS) analyses.

LC-MS analyses were performed on a Waters Acquity ultrahigh-performance liquid chromatography (UPLC) system, coupled to a SYNAPT XS quadrupole time-of-flight (QTOF) high-resolution mass spectrometer with an electrospray ionization (ESI) interface. Lichen specialized metabolites were separated chromatographically using a Kinetex EVO C18 column (150 × 2.1 mm, 1.7 µm). The mobile phase comprised 0.1% formic acid in water (solvent A) and 0.1% formic acid in acetonitrile (solvent B). The gradient elution conditions were as follows: 0-0.5 min, 10% B; 0.5-10 min, a linear gradient from 10% B to 100% B; 10-11 min, 100% B; 11-11.1 min, a linear gradient from 100% to 10% B; 11.1-13 min, 10% B. The flow rate was maintained at 0.45 mL/min, and 5 µL of the sample was injected. Mass spectrometric data for

lichen acids were acquired in negative ion mode with a mass range of 100-1200 m/z. Raw MS data were captured in continuum mode and converted to centroid data using the accurate mass measure function in MassLynx v4.2. Lock mass for negative ion mode was set at 554.2615 m/z. The centroid data were converted to mzML format using MSconvert.

Dataset used to create Figure 1

A) Data used to generate the alluvial plot (Figure 1A)			B) Data used to generate figure 1B; number of genome (cumulative)		
**Year**	**No. of genomes**	**Class**	**Year**	**No. of genomes**	**Class**
2015	5	Lecanoromycetes	2015	5	Lecanoromycetes
2020	17	Lecanoromycetes	2020	22	Lecanoromycetes
2024	402	Lecanoromycetes	2024	424	Lecanoromycetes
2015	0	Lichinomycetes	2015	0	Lichinomycetes
2020	0	Lichinomycetes	2020	0	Lichinomycetes
2024	8	Lichinomycetes	2024	8	Lichinomycetes
2015	175	Saccharomycetes	2015	175	Saccharomycetes
2020	1062	Saccharomycetes	2020	1237	Saccharomycetes
2024	1636	Saccharomycetes	2024	2873	Saccharomycetes
2015	217	Sordariomycetes	2015	216	Sordariomycetes
2020	1896	Sordariomycetes	2020	2122	Sordariomycetes
2024	2706	Sordariomycetes	2024	4817	Sordariomycetes
2015	167	Eurotiomycetes	2015	167	Eurotiomycetes
2020	641	Eurotiomycetes	2020	808	Eurotiomycetes
2024	2225	Eurotiomycetes	2024	2650	Eurotiomycetes
2015	78	Dothideomycetes	2015	78	Dothideomycetes
2020	600	Dothideomycetes	2020	678	Dothideomycetes
2024	1270	Dothideomycetes	2024	1984	Dothideomycetes
2015	59	Leotiomycetes	2015	59	Leotiomycetes
2020	166	Leotiomycetes	2020	225	Leotiomycetes
2024	161	Leotiomycetes	2024	368	Leotiomycetes

Figure 1A was created from Dataset A in the table above using R with the ggplot2 (v3.5.1) and ggalluvial (v0.12.5) packages. Figure 1B was created from Dataset B in the table above using R with the ggplot2 (v3.5.1) package. The numbers refer to the number of genomes freely accessible until August 2024.

Phylogenomic species tree

Genome completeness for all the assemblies (Supplementary Material 1) was assessed using BUSCO (Benchmarking Universal Single-Copy Orthologs) utilizing the Ascomycota or Basidiomycota database (fungi odb_10) as applicable. BUSCO performs quality assessment of genome assemblies by identifying the presence of conserved single-copy orthologous genes and categorizes them as present in single copy, duplicated, fragmented, or missing, based on comparison to a reference set of orthologs from closely related species present in the reference database used. Only genomes with completeness over 90% were analyzed for the presence of ICSs to ensure that incomplete assemblies did not lead to missed detection of the target genes.

We constructed a species tree for 232 taxa using the BUSCO phylogenomics pipeline (https://github.com/jamiemcg/BUSCO_phylogenomics) and IQ-TREE. The BUSCO phylogenomics pipeline employs single-copy BUSCOs to create a concatenated alignment from selected BUSCOs, along with a corresponding character partition file. It processes BUSCO output files to generate concatenated supermatrix alignments and gene trees for BUSCO families (Simão *et al.*, 2015; Manni *et al.*, 2021). The pipeline initially identifies complete, single-copy BUSCO proteins across all input samples and offers flexibility by including proteins that are complete and single-copy in a user-defined percentage of samples, allowing for the accommodation of missing data. After identifying the relevant BUSCO proteins, they are aligned and trimmed to produce individual gene alignments, which are then concatenated into a supermatrix. To infer the maximum likelihood tree, we applied a threshold of 85%, i.e., only the genes present in at least 85% of taxa were selected to create the alignment. Overall, 530 single copy BUSCOs passed this threshold.

The final alignment consisted of 231 sequences with 261,900 columns, 238,050 distinct patterns and 197,299 parsimony-informative sites.

The partition file for the concatenated alignment and supermatrix were used to generate a 1000 bootstrap maximum likelihood phylogenomic tree using IQ-TREE and implementing the LG model of evolution.

Results

Metabolite profiling and molecular networking

The compounds were annotated with high resolution mass spectrometry (HRMS) and verified with literature data. First, HRMS allows the molecular formula prediction using the mass to charge ratio values of deprotonated molecular ions in negative ion mode. Then more detailed structural information is obtained by checking MS fragments, and lichen specialized metabolites usually gives characteristic fragments, particularly for depsides which show fragment ions from the cleavage of ester bonds. Finally, the compound is annotation by comparing its MS data with published metabolite data on the same species and dereplication with online databases, e.g. GNPS. For instance, the metabolite profile of *Pseudevernia furfuracea* is well studied, and we can unambiguously match our annotated lichen metabolites (i.e. atranorin, physodic acid, methyl physodic acid and hydroxyl physodic acid) with published data (Malaspina *et al.*, 2014; Komaty *et al.*, 2016), in terms of molecular formula and MS fragments. We also have jointly developed a lichen metabolite database called Lichendex (submitted Dec 2024), which includes many reference lichen metabolite MS data for fast compound annotation.

Phylogenomic species tree

The 1000 bootstrap maximum likelihood tree was supported, and all the Ascomycota classes formed a supported monophyletic clade. AntiSMASH identified a total of 8,522 biosynthetic gene clusters (BGCs) across the 232 genomes. The number of BGCs varied significantly among fungal classes and taxa (see Supplementary Table S1). Many lichen-forming fungi have taxa have the same or higher number of BGCs than found in closely-related model fungi *Aspergillus nidulans* and *Penicillium subrubescens* (Figure 5, main text).

References

Komaty S, Letertre M, Dang HD, Jungnickel H, Laux P, Luch A, Carrié D, Merdrignac-Conanec O, Bazureau J-P, Gauffre F, *et al.* 2016. Sample preparation

for an optimized extraction of localized metabolites in lichens: Application to Pseudevernia furfuracea. *Talanta* 150: 525–530.

Malaspina P, Giordani P, Faimali M, Garaventa F, Modenesi P. 2014. Assessing photosynthetic biomarkers in lichen transplants exposed under different light regimes. *Ecological Indicators* 43: 126–131.

Manni M, Berkeley MR, Seppey M, Simão FA, Zdobnov EM. 2021. BUSCO Update: Novel and Streamlined Workflows along with Broader and Deeper Phylogenetic Coverage for Scoring of Eukaryotic, Prokaryotic, and Viral Genomes. *Molecular Biology and Evolution* 38: 4647–4654.

Simão FA, Waterhouse RM, Ioannidis P, Kriventseva EV, Zdobnov EM. 2015. BUSCO: assessing genome assembly and annotation completeness with single-copy orthologs. *Bioinformatics* 31: 3210–3212.
